# The Medicine Wheel as a public health approach to lifestyle management interventions for indigenous populations in North America

**DOI:** 10.3389/fpubh.2024.1392517

**Published:** 2024-07-19

**Authors:** Tammy Greer, Jennifer L. Lemacks

**Affiliations:** ^1^Mississippi INBRE Community Engagement and Training Core, Center for American Indian Research and Studies, Telenutrition Center, School of Psychology, The University of Southern Mississippi, Hattiesburg, MS, United States; ^2^Mississippi INBRE Community Engagement and Training Core, Telenutrition Center, School of Health Professions, College of Nursing and Health Professions, The University of Southern Mississippi, Hattiesburg, MS, United States

**Keywords:** Native American, lifestyle management, indigenous models, disease prevention, health behaviors

## Abstract

This analytic essay intends to elevate Medicine Wheel, or generally “four directions” teachings, to encourage a more comprehensive alignment of lifestyle intervention components with traditional ecological knowledge systems of Indigenous cultures in North America. North American Medicine Wheels provided people with a way to orient themselves both within their traditional belief systems and to the seasonal changes in their areas, improving survivability. The wheel or circle is a sacred symbol, indicating the continuity and perpetuity of all of life. The four directions are iconized in many Indigenous cultures across North America with different directions representing different aspects of our world and of ourselves, different seasons of the year and of our lives, different beings of the earth and tribes of humans with a balance among those necessary for health and wellbeing. In the context of public health, teachings of the four directions warn that a lack of balance limits our ability to achieve optimal health. While there is much public health success in lifestyle interventions, existing practice is limited by a siloed and one size fits all approach. Medicine Wheel teachings lay out a path toward more holistic and Indigenous-based lifestyle intervention that is modifiable depending on tribal teachings and needs, may appeal to a variety of Indigenous communities and is in alignment with health behavior change theory. It is a public health imperative that lifestyle management interventions are fully optimized to rigorously determine what can be achieved when interventions are implemented in a holistic and Indigenous-based manner, and in alignment with an Indigenous model of health. This more complete alignment would allow for a stronger foundation to further explore and develop social determinants (i.e., housing, employment, etc.) and structural intervention enhancements to inform public health practice and promote health equity.

## 1 Introduction

**Figure 1:**
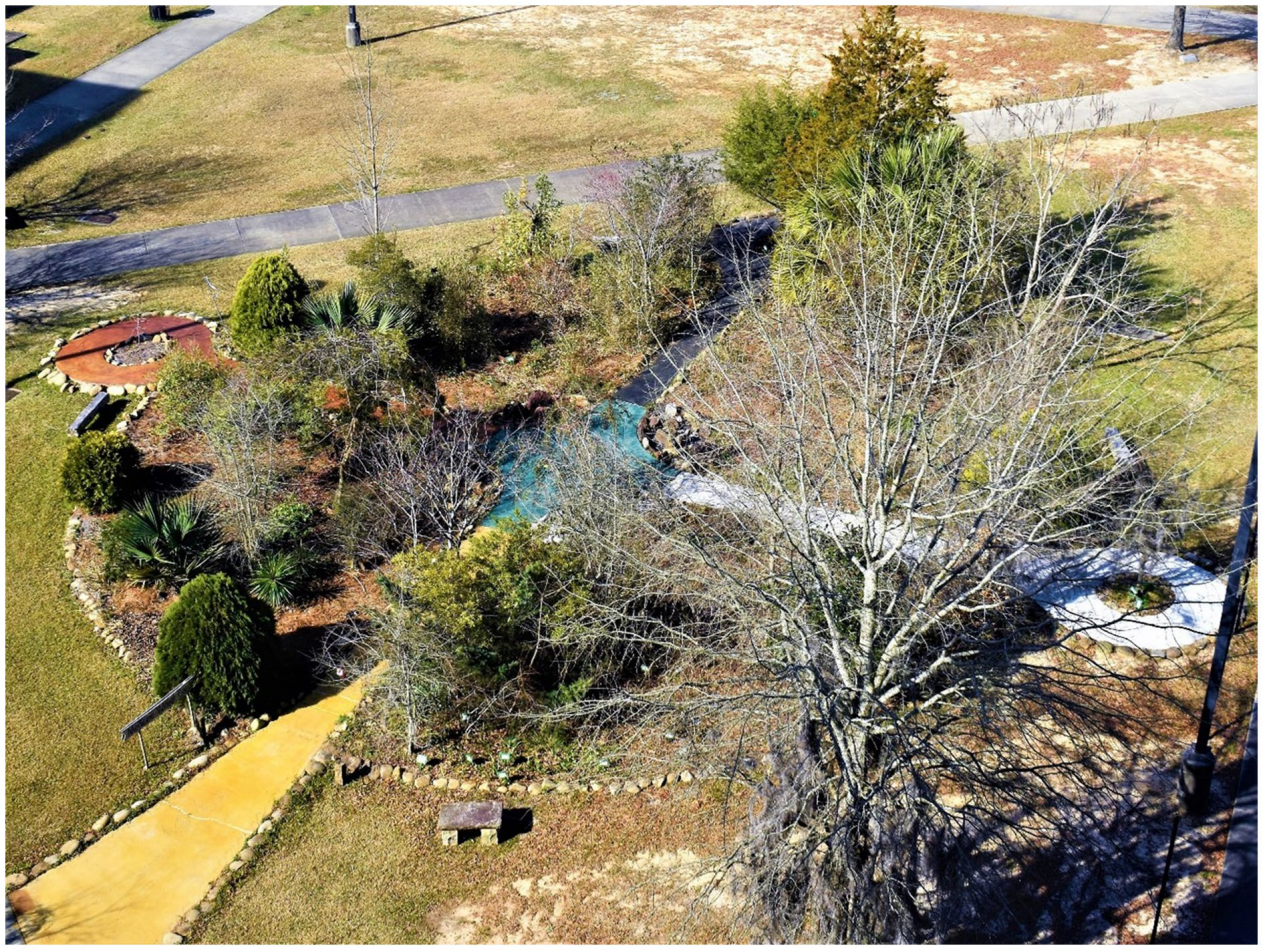
The Southern Miss Medicine Wheel garden. The garden is in Hattiesburg, Mississippi on The University of Southern Mississippi’s campus and was established in 2005. It is a modern-day representation of Medicine Wheel teachings and knowledge, and features plants native to the Deep South.

Medicine Wheels (more northern) and earthen mounds (more southern) are scattered across the Americas. Rock cairns inside Medicine Wheels and mound orientations were designed to align with solstices, stars, and important geographic locations (1, 2). These astrological and geographic orienting systems provided tribal people with a way to orient themselves within their traditional belief systems and communities, to seasonal changes and to important geography (1). Rock cairns and mound structures throughout the Americas were placed to touch the sun on the days of winter or summer solstices, to orient Indigenous people to when the days were to become longer or shorter, predicting the coming of summer or winter (1, 2). Medicine Wheel (Figure 1) orientations toward seasons, the natural ebb and flow of light and dark, the cyclical nature of everything, provided, as well, an understanding of where humans were in that cycle, allowing for necessary preparations for what was to come and, improving predictability and, ultimately, survivability. For most North American Indigenous cultures, the circle is a sacred symbol, and the four directions are iconized with stone structures, painted on rocks, cave walls, and shells, indicating these are ancient and meaningful symbols across the Americas. Teachings have been developed around the symbol of the circle surrounding the paths in four directions that comprise the sacred Medicine Wheel. In general, contemporary Medicine Wheel teachings speak to a holistic understanding of the aspects of our own human natures, the natures of other beings, and that of our world, and the balance of those aspects that facilitates navigation of challenging environments. This traditional paradigm has the potential to advance lifestyle interventions that address preventable chronic disease disparities that continue today among Indigenous populations in North America, especially in the Deep South.

Diet and physical activity lifestyle modification programs mitigate chronic disease risk and promote quality of life by improving associated cardiometabolic outcomes (weight, blood pressure, glucose, and lipid levels). Standard lifestyle modifications include enhancing self-efficacy for diet/physical activity behavior changes and improving healthy behaviors. The National Diabetes Prevention Program (NDPP) is the most renowned standard lifestyle modification program showing that behavior modification without medication was more effective at diabetes prevention compared to medication alone with sustained outcomes over time (3). As an example of a public health success, the NDPP has been adopted and supported for implementation across the US and translated to Indigenous populations across the US (Special Diabetes Program for Indian Diabetes Prevention; SDPI-DP). The SDPI-DP and NDPP outcomes in 80 tribes had similar post-intervention cardiometabolic improvements (4). Only one of those 80 tribes was a Deep South tribe. Despite significant post-intervention improvements in health outcomes, Jiang et al. (4) reported large dropout rates attributing participant-level factors of retention failure to a lack of family support, becoming diagnosed with diabetes, and self-reported disinterest in the program (4, 5). Even with successes in the adoption of SDPI-DP across the nation, health disparities continue to exist among Tribal Nations in the Deep South. More fully integrating traditional teachings that have survived for centuries into culturally-tailored lifestyle interventions may be the solution to address health disparities in Tribal Nations. Thus, the purpose of this analytic essay is to elevate Medicine Wheel teachings to encourage a more comprehensive alignment of lifestyle intervention components with a traditional ecological knowledge system of North American Indigenous cultures.

## 2 Medicine Wheel as behavior change theory to guide better health

Medicine Wheel teachings are indigenous teachings used by different tribes to serve numerous purposes, often focused on diverse areas of health and wellness. Medicine Wheel teachings remind us that there are four aspects to our world (fire, earth, water, and air), four seasons (spring, summer, autumn, and winter), four aspects to our humanity (spiritual, emotional, physical, and intellectual) and four stages of our lives (birth, youth, adult/elder, and death) (6). Just as one season is not more important than another, it is also true that no one aspect of ourselves or stage of our lives is more important than the others. Optimal functioning of ourselves and our world, the health and wellness of our spirits, emotions, bodies, and minds depends on a balance of all aspects with consequences resulting from imbalance. From Medicine Wheel teachings, we know that individuals grow and change as they develop, only to revisit areas of themselves and grow and change more at each pass on the wheel. Deaths are endings and as is true with all endings – the end of our bodies, friendships, employment, education, projects, laws, and even stars – are followed by new life. The teachings are extensive, and regarding health, there is much in common between ancient teachings and health behavior change theories that have emerged in the last century.

Recognizing the complexity of changing nutrition and physical activity behaviors, wellness theory suggests that there are six-to-eight mutually interdependent dimensions of wellness that contribute to healthy living (7). Hettler (7) established the foundational six dimensions of wellness to include emotional, occupational, physical, social, intellectual, and spiritual dimensions. Recognizing that behavior change is multi-faceted and involves multiple levels of influence, the socioecological model (SEM) suggests multiple interdependent factors that interact within and across levels to influence human behavior (8). The SEM was adapted for health promotion and disease prevention to recognize multiple levels of influence on health behaviors (8). The levels of the model include intrapersonal/individual, interpersonal, institutional/organizational, community, and public policy factors. Both wellness and SEM theories suggest that well-being is achieved and programs are most effective when they are designed to address multiple levels of influence on health behaviors. From their inception, standard lifestyle interventions have historically focused on the physical and intellectual dimensions of health at the individual level. For example, increasing self-efficacy to eat healthier is an intellectual improvement, whereas healthier dietary behaviors is a physical improvement; both are intrapersonal factors. However, it is becoming more obvious, and in alignment with Medicine Wheel teachings and current health behavior theories, that targeting multiple dimensions and levels ensures a more comprehensive health promotion or disease prevention program. The Medicine Wheel offers ancient teaching and essentially behavioral theory may guide the development of evidence-based health interventions in the same way that modern theory is used.

## 3 Integral Medicine Wheel domains of health to explore

Whereas physical and intellectual domains are important to achieve optimal health, other determinants have also emerged as influencers of health behavior among Indigenous populations in North America. Previous findings support the importance of the spiritual and social domains as well as the family SEM level to facilitate improved lifestyle management among Indigenous populations in North American. The SDPI-DP data demonstrated that spirituality was negatively correlated with weight at baseline (9). Generally, Indigenous populations in North American have exhibited fatalistic views (10) including a resignation to acquiring diseases like diabetes (10, 11), that can be addressed in the spiritual domain. This spiritual aspect is likely a conditioned response and a resultant cultural norm following repeated exposure to premature death and loss within North American Indigenous communities (12). Various types of fatalism have been measured among other populations as a barrier to behavior change, and fortunately, prior research (13) has shown that it is possible for health interventions to reduce fatalistic thinking in minority populations.

Research also supports that family is a potentially important contributor to the attainment of positive health outcomes among Indigenous populations in North America, indicating the importance of the social domain for this population. Jiang et al. (4, 5) reported that lack of family social support was a major predictor of SDPI-DP dropout and linked to less weight lost post-intervention (9). In contrast, family support of dietary behaviors has been shown to improve diabetes control among Navajo members (14). Jiang et al. (5) also reported in a later study that participants who lacked family support were at greater risk for not attending intervention sessions and loss to follow up, indicators of short- and long-term retention failure. Positive family social support was a predictor of greater weight loss over the course of the intervention, whereas psychosocial factors such as coping skills and trauma exposure were not predictors (5). Studies that have implemented and assessed a socially and culturally enhanced diabetes management intervention among Indigenous populations in North America are rare and what is available is problematic with regard to making comparisons to the original SDPI-DP to ascertain any added benefits. Evidence is insufficient to support the contribution of social and cultural enhancements to a standard intervention approach and resulting public health practice implications for Indigenous populations in general and in the Deep South.

While it is well supported among Indigenous and across populations that family support is critical for lifestyle change, there are some limitations regarding translational evidence for lifestyle interventions. For example, in a review (15) of 26 family-based interventions for adults with diabetes, there was evidence that family-based interventions resulted in self-reported psychosocial (i.e., perceived social support or self-efficacy, or diabetes self-care) improvements; however, it was difficult to ascertain any clinical improvements across studies due to heterogeneity of study design, outcomes, interventions, and level of family involvement. Included in this review were the Family Education Diabetes Series (FEDS) trial (16, 17) and the Strong in Body and Spirit lifestyle intervention (18) that implemented family-based interventions in Indigenous communities in North America. Gilliland et al. (18) reported hemoglobin A1c% increased among all three treatment groups, albeit the increase was smaller and statistically insignificant in the family and friends group and the one-on-one group compared to usual care. The FEDS trial did show significant improvements in weight, blood pressure, and A1c at three- and six-month intervals in 36 participants (16); participants also reported that the social support and group-orientation of the program were the most salient components of the intervention (17). Comparing these two studies in Indigenous populations in North American, the dilemma of study heterogeneity reported by Baig et al. (17) becomes clear. A more recent review of family-based randomized control trials among adults with type 2 diabetes showed moderate heterogeneity across trials (*n* = 18) and reported similar issues related to variable family involvement across trials (19). However, results did show significant improvements in A1c levels with greater improvements in developing areas compared to developed countries (19).

A PubMed search (12/8/2023) of terms [(family-focused) or (family-based) AND intervention Native American] produced 42 results and only three distinct family-based interventions (20–22). The Healthy Children, Strong Families intervention showed improvements in nutrition, physical activity and body weight among Indigenous families with young children (20). A second trial, Together on Diabetes Trial, enrolled 255 adolescents and 223 of their “support persons” in a lifestyle intervention program to reduce type II diabetes risk among adolescent youth but did not report post-intervention results (21). The Shadow Project was an intervention aimed to engage families to reduce substance use behaviors among adolescents with no follow up results reported (22). Although it is clear that the social domain is important to promote lifestyle behavior change, the question remains, how do we operationalize the social domain toward the implementation and dissemination of evidence-based lifestyle interventions into public health settings? The answer to this question may have great implications for the health of Indigenous populations in North American and broader populations.

Similarly, there is limited evidence for effective spiritual adaptations to lifestyle interventions that consider Indigenous worldviews. From our own past work, an Indigenous student who was a member of a tribe located in the Deep South conducted a systematic literature review following the PRISMA guidelines (23). The work evaluated peer-reviewed literature published over the past 20 years that was explicit to culturally informed type 2 diabetes (T2DM) prevention/management interventions in Indigenous populations in the US. PRISMA guidelines were followed and search terms included (Native American AND cultural intervention AND type 2 diabetes) and publication dates from 2009 to 2021. Studies were examined for standard or enhanced cultural adaptations, and classified into one of those two groups, and had to include effectiveness outcomes. Since a 2008 review (24), only five studies were found that met inclusion criteria of having a culturally informed intervention that addressed T2DM among Indigenous populations in the US (25–29) out of 205 articles screened; 16 of the 205 were further assessed for eligibility with 11 excluded to yield the five articles due to not addressing T2DM (*n* = 3), not being culturally-informed (*n* = 3), not being an intervention (*n* = 3), or only describing methods with no available published results (*n* = 2). We consider standard cultural adaptations to include representation of Indigenous people, language, foods, and resources in curriculum and materials and enhanced cultural adaptations to include more robust cultural elements, such as Medicine Wheel concepts, talking circles, and digital storytelling. Three interventions (26, 28, 29) were categorized as standard adaptations and two interventions (25, 27) were classified as enhanced cultural adaptations. Post-intervention outcomes varied widely with some significant post-intervention changes to weight, diet, and physical activity noted compared to comparison/control groups (25, 28, 29). None of the tribal populations represented were in the Deep South. Results also did not indicate what cultural adaptations could add to standard lifestyle management interventions. Thus, the success rate of culturally informed interventions is not yet supported in the literature and more vigorous research is needed to determine the added benefit of cultural components to standard lifestyle interventions.

## 4 Toward holistic lifestyle management implementation

**Figure 2:**
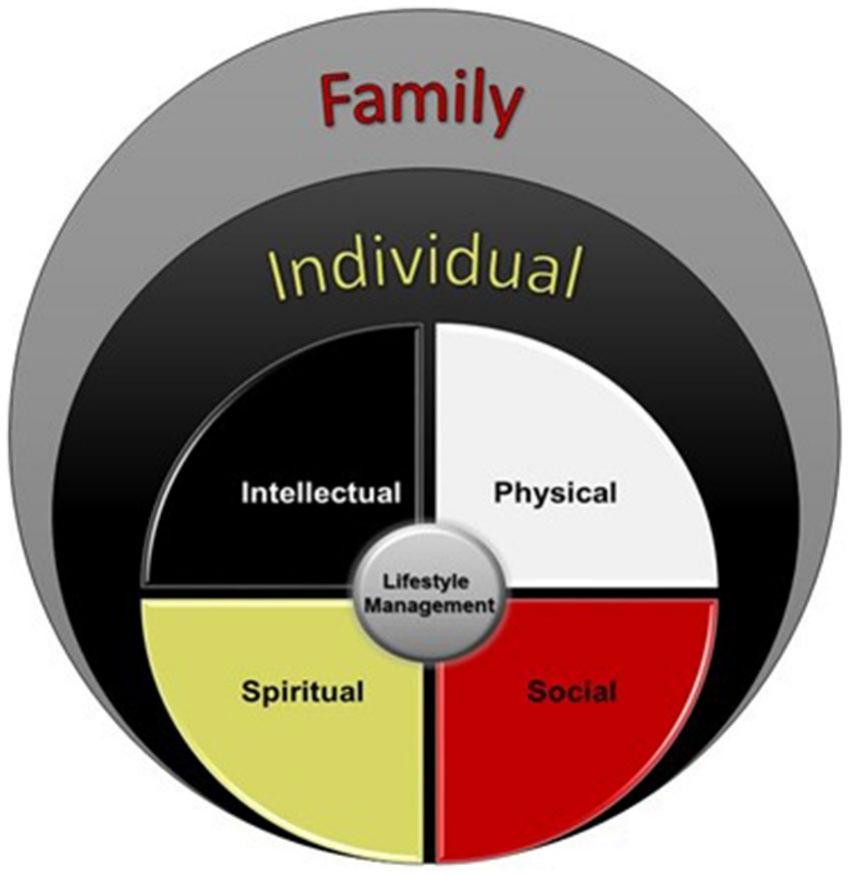
A multi-domain and multi-level approach in alignment with the Medicine Wheel. The Okla Achokma model examines the implementation of a more holistic approach that addresses four domains and two levels that have been identified as key aspects of lifestyle management among Native Americans in the Deep South. The four critical domains of lifestyle management are the intellectual, physical, spiritual, and social domains and addressed at the individual level. The family is an additional level that is posited to serve as a support system for the individual. This model allows for expansion to consider additional levels of influence, such as community or organizational levels.

Progress in lifestyle intervention research and promising interventions with enhanced cultural adaptations are beginning to emerge for Indigenous communities (30). Holistic and multi-level approaches that consider various health domains are needed to advance standard lifestyle interventions as mortality disparities persist, with retention and participation of Indigenous populations in the Deep South under-examined and likely poor based on available evidence. To advance lifestyle interventions, researchers and practitioners must first identify population specific critical domains of health and effective evidence-based strategies to address them in collaboration with the intended population. Research has supported that Indigenous populations prefer interventions designed for their specific culture/tribe and with their participation in the process (31, 32). For Indigenous populations in the Deep South, previous findings and our own preliminary works suggest that the social and spiritual domains and the family SEM level have implications for cultural and social enhancement of lifestyle management interventions (10, 12, 33). The spiritual domain focuses on finding cultural value, traditional value and connection with being a healthy person, whereas the social domain enhances connections with community and within family. Our Okla Achokma model (Figure 2) is an example of how we have conceptualized the factors to be addressed by lifestyle management in a holistic manner that honors both traditional knowledge and rigorous, theory-guided health behavior science.

The Okla Achokma model respects the interrelationship among the four aspects of being human including the intellectual, physical, spiritual, and social domains. Lifestyle management programs address the intellectual aspect by improving knowledge and understanding of the relationship between lifestyle choices, such as diet and physical activity, and disease. The spiritual aspect is addressed by the removal of existential barriers such as fatalism through re-alignment with traditional values such as respect for what our bodies need to be healthy, courage to make hard and healthy choices, and humility to see beyond ourselves and consider how our changes will affect the health and wellbeing of the next generation. The social aspect is addressed by considering that individuals are only one part of a web of social connections and social support from family/community is integral to achieve and sustain healthy behaviors. The physical aspect involves how we nourish and tend our bodies with diet and physical activity behaviors. Medicine Wheel teachings indicate that calling each of these aspects of ourselves into service, in a balanced way, will naturally result in becoming Okla Achokma (“healthy people” in the Choctaw language) who live long and healthy lives. Testing this Indigenous model of health and exploring the added benefit of holistic health interventions in a manner that acknowledges traditional ways of thinking is urgently needed.

While this model aims to maximize interventions targeting the inter- and intra-personal levels, this is the first step toward building a holistic, optimized intervention model. As SEM suggests, which is reverberated throughout the literature, numerous structural factors are critical to addressing health disparities in Tribal populations, including health and social policies and guidelines that govern how resources are distributed to Tribes and may contradict the sovereignty of those nations. As the Medicine Wheel suggests, these factors (from interpersonal to structural factors) are interrelated, and we could expect synergistic effects with changes at multiple levels. Thus, the Okla Achokma model is an example of a first step to optimize current evidence-based lifestyle interventions. The optimization of these interventions with enhanced cultural adaptations will define the most that can be gained from interventions that target the inter/intra-personal levels. Knowing the maximum gains from inter/intra-personal interventions will provide a solid foundation for the next step to examine the added benefit of interventions aimed at organizational, environmental, and policy levels.

## 5 Conclusion and charge for public health research

The NDPP and SDPI are well-adopted across the US as successful public health efforts and there is much related research being done to build on this success. Multiple integral domains of health for Indigenous populations in North America, especially those who are underrepresented, underserved, and underreached in the literature (i.e., Tribal Nations in the Deep South), must be considered to advance the science in this public health area. It is imperative that lifestyle management interventions are fully optimized to rigorously determine the most that can be achieved when interventions are implemented in a holistic and enhanced culturally tailored manner, in alignment with an Indigenous model of health. This advancement would allow for a stronger foundation to further explore and develop social determinants (i.e., housing, employment, etc.) and structural intervention enhancements to inform public health practice and promote health equity. Medicine Wheel teachings indicate that all influencers of health are intertwined and interconnected, and therefore, the solution is not behavior, biology, social or environmental conditions alone. The solution is in addressing the complex intertwined multitude of factors that, altogether, perpetuate health disparities. It is time to depart from the silo and one-size-fits all approach and advance the health sciences to meet this level of complexity, for the sake of our neighbors.

## Author contributions

TG: Conceptualization, Funding acquisition, Writing – original draft, Writing – review & editing. JL: Conceptualization, Funding acquisition, Writing – original draft, Writing – review & editing.
